# Neurological, Radiological, Visual, and Auditory Findings in Children with Intrauterine Exposure to the Zika Virus

**DOI:** 10.3390/v17020238

**Published:** 2025-02-09

**Authors:** Marlos Melo Martins, Andréa Bittencourt Guastavino, Maria Clara de Magalhães-Barbosa, Maria Helena de Magalhães-Barbosa, Cristiane Fregonesi Dutra Garcia, Bárbara Karine Gonet Amaral, Annamaria Ciminelli Barbosa, Halina Cidrini Ferreira, Jaqueline Rodrigues Robaina, Mariana Barros Genuino de Oliveira, Fernanda Freire Tovar-Moll, Roberto de Andrade Medronho, Antonio José Ledo Alves da Cunha, Joffre Amim, Arnaldo Prata-Barbosa

**Affiliations:** 1 Division of Pediatric Neurology, Martagão Gesteira Institute of Childcare and Pediatrics (IPPMG), Federal University of Rio de Janeiro (UFRJ), Rio de Janeiro 21941-912, RJ, Brazil; marlosmartins@me.ufrj.br; 2 Neonatal Unit, Maternity School Hospital, Federal University of Rio de Janeiro (UFRJ), Rio de Janeiro 22240-001, RJ, Brazil; abguastavino@gmail.com; 3 Postgraduate Program, Maternity School Hospital, Federal University of Rio de Janeiro (UFRJ), Rio de Janeiro 22240-001, RJ, Brazil; joffre@me.ufrj.br; 4 Department of Pediatrics, D’Or Institute for Research and Education (IDOR), Rio de Janeiro 22281-100, RJ, Brazil; mariaclara.magalhaes@idor.org (M.C.d.M.-B.); jaqueline.robaina@idor.org (J.R.R.); mariana.genuino@idor.org (M.B.G.d.O.); antonioledo@yahoo.com.br (A.J.L.A.d.C.); 5 Phonoaudiology Unit, Maternity School Hospital, Federal University of Rio de Janeiro (UFRJ), Rio de Janeiro 22240-001, RJ, Brazil; mariahelena_barbosa@yahoo.com.br (M.H.d.M.-B.); crisfdgarcia@gmail.com (C.F.D.G.); 6 Department of Phonoaudiology, School of Medicine, Federal University of Rio de Janeiro (UFRJ), Rio de Janeiro 21044-020, RJ, Brazil; 7 Department of Ophthalmology, Maternity School Hospital, Federal University of Rio de Janeiro (UFRJ), Rio de Janeiro 22240-001, RJ, Brazil; barbaragonet@yahoo.com.br; 8 Department of Ophthalmology, Gaffree and Guinle University Hospital, Federal University of the State of Rio de Janeiro (UNIRIO), Rio de Janeiro 20270-004, RJ, Brazil; annamariaciminelli@hotmail.com; 9 Department of Physiotherapy, Maternity School Hospital, Federal University of Rio de Janeiro (UFRJ), Rio de Janeiro 22240-001, RJ, Brazil; halinacidrini@me.ufrj.br; 10 Department of Radiology, D’Or Institute for Research and Education (IDOR), Rio de Janeiro 22281-100, RJ, Brazil; fernanda.tovarmoll@idor.org; 11 Department of Epidemiology and Public Health, School of Medicine, Federal University of Rio de Janeiro (UFRJ), Rio de Janeiro 21044-020, RJ, Brazil; robertoamedronho@gmail.com; 12 Department of Pediatrics, School of Medicine, Federal University of Rio de Janeiro (UFRJ), Rio de Janeiro 21044-020, RJ, Brazil; 13 Department of Gynecology and Obstetrics, School of Medicine, Federal University of Rio de Janeiro (UFRJ), Rio de Janeiro 21044-020, RJ, Brazil

**Keywords:** Zika virus, microcephaly, infantile development, radiological abnormalities, visual disorders, auditory disorders

## Abstract

This study aims to describe neurological, visual, and auditory findings in children whose mothers had confirmed Zika virus (ZIKV) infection during pregnancy, with most of these children not presenting congenital microcephaly; Methods: an observational, longitudinal, and prospective study was conducted in Rio de Janeiro, Brazil, from March 2015 to January 2017, involving children with in utero exposure to Zika virus, following from birth up to 30 months of age. Results: Of the 2882 pregnant women admitted, 116 had a suspected ZIKV infection, of whom 33 had laboratory confirmation. Only one child presented with congenital microcephaly. Despite this, neurodevelopment delay was observed in 36.4% of children evaluated, radiological abnormalities in 29.1%, auditory abnormalities in 8.3%, and ophthalmological abnormalities in 10%. Conclusions: Newborns of mothers with confirmed ZIKV infection during pregnancy may present with varying degrees of visual, auditory, and neurological impairment, despite the presence of congenital microcephaly.

## 1. Introduction

Congenital Zika Syndrome (CZS) was first recognized in Brazil during the 2015–2016 Zika virus outbreak, which coincided with a dramatic increase in cases of microcephaly in newborns, particularly in the northeast region of the country [1]. This condition, a direct result of Zika virus infection during fetal development is now understood as a spectrum of abnormalities, including not only microcephaly, but also profound neurological, visual, and auditory impairments [2,3,4,5]. These findings underscore the substantial individual, familial, and global health implications of CZS [6].

Although microcephaly at birth was initially considered the hallmark of CZS, subsequent research has revealed that this is not an essential feature. Many children exposed to the Zika virus in utero exhibit a range of neurological and sensory deficits, despite having a normal head circumference at birth [7,8,9]. This highlights the need for further investigation into the broader clinical spectrum and natural history of the syndrome, particularly in children without microcephaly.

There remains limited information on the long-term growth and neurological outcomes of children with CZS, especially in those without overt microcephaly. Few studies have explored the outpatient follow-up and developmental trajectories of affected children, making it critical to characterize their neurological, visual, and auditory manifestations. This study aims to address this gap by focusing on children born to mothers with confirmed Zika virus infection during pregnancy, most of whom did not present with microcephaly at birth.

## 2. Materials and Methods

### 2.1. Study Design and Setting

An observational, longitudinal, and prospective study was conducted at the Maternity School Hospital of the Federal University of Rio de Janeiro, Brazil (ME-UFRJ). The source population consisted of all pregnant women who agreed to participate in this study from 3 December 2015 to 31 January 2017 and who had confirmed infection by the Zika virus during pregnancy through (1) detection of viral RNA by the reverse transcriptase reaction followed by polymerase chain reaction (RT-PCR) in any biological sample (blood, urine, amniotic fluid, placental fragment, etc.), and/or (2) positive IgM serology for the Zika virus, confirmed by an increase in IgG neutralizing antibodies ≥ 4 times concerning the titers of these antibodies against the dengue virus (to avoid the potential for cross-reactivity with ZIKV antibodies due to its endemic pattern in Rio de Janeiro, which could have led to false-positive results). The test was considered inconclusive if IgG against the Zika virus was <4 times higher than that of IgG against the dengue virus. The study population consisted of children born to this source population, included consecutively, who were monitored at the ME-UFRJ follow-up clinic from birth to 30 months of age.

Pregnant women with suspected infection or those with microcephalic fetuses were selected for laboratory investigation. Blood collection for RT-PCR was performed up to the 5th day from the onset of symptoms but could be extended up to the 14th day (to minimize the risk of false-negative results, considering the time of Zika virus viremia in different sample types). A urine sample for RT-PCR was collected up to the 28th day. In addition, blood samples from pregnant women were collected between the 7th and 14th days to test for immunoglobulin M (IgM) and immunoglobulin G (IgG). Investigation for dengue and chikungunya (to exclude the possibility of cross-reactions with immunological tests), in addition to other diseases in the TORCH group (toxoplasmosis, rubella, cytomegalovirus, human immunodeficiency virus, VDRL and herpes simplex), was also performed. All material was sent to the central laboratory of the Health Department of the State of Rio de Janeiro, and the pregnant women began to be monitored in the prenatal care of ME-UFRJ. At delivery, umbilical cord blood was collected for RT-PCR for ZIKV.

### 2.2. Newborn Monitoring in the Immediate Neonatal Period

For all confirmed cases of newborns with intrauterine exposure to the Zika virus, laboratory, imaging, and other screening tests were performed: (1) RT-PCR for Zika virus, Chikungunya, and dengue in the cord blood and cerebrospinal fluid of newborns with congenital microcephaly; (2) Serology (IgM and IgG) for Zika virus, Chikungunya in peripheral blood on the fifth day of life; (3) Collection of peripheral blood on the fifth day of life for TORCH screening; (4) Transfontanellar ultrasound with Doppler was performed in the first week of life through the anterior and temporal fontanelle, performed with a Philips HD7 XE ultrasound device, with a C8-5 mini convex probe (Philips, Eindhoven, The Netherlands); (5) Neonatal Hearing Screening: Transient Evoked Otoacoustic Emissions (TEOAE) was performed in the first 24 to 48 h of life, following the guidelines of the Neonatal Hearing Screening Care Guidelines of the Ministry of Health [4]. For preterm infants, Brainstem Auditory Evoked Potentials (BAEP) was performed at the corrected gestational age of 34 weeks to avoid erroneous interpretations secondary to prematurity or immaturity of the auditory system. All exams were performed by the same examiner using Otoport Lite OEA DP+TE (Otodynamics, Hatfield, England), which uses non-linear click stimulus (which covers frequencies from 1 to 5 KHz), with an equivalent peak intensity of approximately 84 dB SPL (decibels sound pressure level). An acceptable stimulus stability of 70% or greater was considered for the exam to be valid, and the standard noise rejection value was 47 dBNPS. For the test to be considered positive (TEOAE present), otoacoustic emissions had to project above the noise level in most of the frequency bands covered; that is, the signal-to-noise ratio had to be greater than or equal to 3 dB and the reproducibility by frequency band or overall, greater than or equal to 50%; (6) Neonatal Eye Screening was performed by a trained pediatrician. Once any alteration was detected, the neonate was referred for evaluation by an ophthalmologist and, if necessary, directed to a specialized rehabilitation service (Specialized Center for Rehabilitation with visual modality or Visual Rehabilitation Unit) or even to specialized ophthalmology services according to the “Guidelines for Eye Health Care in Childhood” of the Ministry of Health [10,11,12].

### 2.3. Outpatient Monitoring

General care for the newborn followed the standards recommended by the Ministry of Health [12], and the first consultation with the pediatrician was preferably during the first week of life. The remaining consultations occurred monthly in the first 6 months, bimonthly from 6 to 12 months, quarterly from 12 to 18 months, and quarterly from 18 months of life. Referral to specialized services was made whenever considered necessary. The intervals between consultations with the various specialists were determined individually in case functional impairment was confirmed.

Global neurodevelopment was assessed longitudinally by a pediatrician, pediatric neurologist, and physical therapist. The assessment by the neuropediatrician occurred with a 3-month interval between appointments, reducing the interval when the specialist deemed it necessary. A complete neurological examination was performed, and the Gesell neurodevelopment test was applied, corresponding to a direct assessment and observation of the quality and integration of behaviors. The analysis categories of this scale refer to the following areas: Adaptive Behavior, Gross Motor, Fine Motor, Language, and Personal–Social Behavior [13].

All patients were referred for cranial computed tomography (CT) and/or encephalic magnetic resonance imaging (MRI), even those without alterations initially revealed by transfontanellar ultrasound and with normal head circumference, undergoing a more accurate study of brain anatomy. The images were acquired in the standard medical imaging format protocol 3.0—Digital Imaging and Communications in Medicine (DICOM)—and distributed to a specific server for later analysis. Each participant underwent a battery of neuroimaging performed on a 16-channel multi-slice CT scanner (Siemens, Munich, Germany) and a 1.5T MRI magnet (General Electric, Boston, MA, USA). Anatomical MRI images were obtained with the following multiplanar pulse sequences: T1-weighted volumetric sequence, T2-weighted turbo spin-echo, FLAIR (Fluid attenuated inversion recovery), and diffusion.

The fundoscopy (FO) or retinal mapping was performed by the same ophthalmologist and occurred at three different times. The first evaluation was conducted in the first week of life, the second at 30 days, and the third at 6 months to identify possible changes compatible with congenital infection.

In most of the infants evaluated, a new evaluation with BAEP was repeated between 18 and 24 months of age (BAEP-a: Automated click BAEP; BAEP-neuro: Click BAEP for neurodiagnosis; BEAP-SF: Frequency-specific BAEP).

### 2.4. Ethical Aspects

This study was approved by the Research Ethics Committee of Maternity School Hospital, from the Federal University of Rio de Janeiro, Brazil (protocol code CAAE: 55465616.0.0000.5275, Opinion Number: 1,516,904, as of 27 April 2016). All participants signed a written informed consent form, agreeing to be included in the study along with their children.

## 3. Results

During the study period, 2882 pregnant women were admitted, of whom 116 (4%) were suspected of having ZIKV infection, and 33 had this infection confirmed. Among these, there was one twin pregnancy and three fetal deaths, resulting in 31 newborns being included in this study. Of the 33 pregnant women included, 31 (93.9%) had diagnostic confirmation of ZIKV infection through a positive RT-PCR test, and two (6.1%) were confirmed through positive IgM serology for ZIKV. Notably, the two women with IgM-based diagnostic confirmation were simultaneously IgM-negative for dengue virus, further supporting the diagnosis of recent ZIKV infection in these patients (Table S1 in the Supplementary Material). The main clinical and epidemiological characteristics of the pregnant women included in this study are presented in Tables S2 and S3 (Supplementary Material).

Laboratory screening for TORCH group infections was conducted as part of the prenatal care, for most patients eligible for this study (Table S4, Supplementary Material). Two patients had indeterminate IgM serology for herpes virus. Serological testing and RT-PCR for herpes simplex, imaging, and fundoscopy (FO) were performed in both newborns, and congenital infection was ruled out. Two pregnant women had syphilis during pregnancy; they were treated, and congenital syphilis was ruled out in their newborns. One pregnant woman was a carrier of the human immunodeficiency virus diagnosed before pregnancy. This patient received antiretroviral treatment during pregnancy and intrapartum, and her newborn received chemoprophylaxis for HIV exposure. None of the tests performed in newborns to investigate congenital infection in the TORCH group showed positive IgM for toxoplasmosis, rubella, CMV, or herpes (Table S5, Supplementary Material).

Of the 31 newborns, 27 (87.1%) were born at a gestational age between 37 and 41 weeks, 3 (9.7%) were born between 28 and 32 weeks, and 1 (3.2%) was born at a gestational age between 33 and 36 weeks, resulting in a total of 12.9% premature births. Notably, there were no births with a gestational age of less than 28 weeks. Twenty-two (71%) were female. One newborn (3.2%) was classified as small for gestational age, and five (16.1%) were admitted to the neonatal ICU.

Only one newborn (3.2%) had microcephaly at birth (head circumference of 28.5 cm), classified as severe microcephaly. His exposure occurred in the first trimester of pregnancy. This patient underwent a lumbar puncture on the second day of life. The results of the cellularity and biochemistry of the cerebrospinal fluid were normal, and the RT-PCR for ZIKV was negative. The fetus identified as microcephalic in the obstetric ultrasound suffered a spontaneous abortion. No newborn developed microcephaly in the postnatal period, no other associated malformations were identified in the group of children studied, and there were no deaths in the neonatal period. The clinical and epidemiological characteristics of the newborns included in this study can be found in Tables S6 and S7 (Supplementary Material).

Of the 31 children in this study (from 30 pregnant women, due to 1 twin pregnancy within the sample), 4 patients were lost to follow-up and 27 (87.1%) were monitored in the outpatient clinic throughout the study period.

### 3.1. Auditory Evaluation

All children underwent some hearing assessment during the study period (TEOAE: Transient Evoked Otoacoustic Emissions; BAEP: Brainstem Auditory Evoked potentials; BAEP-a: Automated click BAEP; BAEP-neuro: Click BAEP for neurodiagnosis; BEAP-SF: Frequency-specific BAEP). Thirty-one children (100%) underwent TEOAE. Only 3 children (9.7%) underwent all hearing assessment tests, and 21 (67.8%) underwent TEOAE + BAEP. The results of the hearing tests performed for each child tested can be found in Table S8 (Supplementary Material). Only one child (3.2%) presented alterations in TEOAE (failure in the left ear), but the subsequent hearing assessment with neuro BAEP was normal. No child presented alterations in BAEP a, one (8.3%) presented alterations in neuro BAEP, and no child who underwent BAEP FS presented alterations in the examination.

### 3.2. Ophthalmological Evaluation

Thirty children (96.8%) underwent retinal mapping exams in the first week of life. The results of the retinal mappings performed can be found in Table S9 (Supplementary Material). Three children (10%) presented some alteration in the first fundus exam, with two of them maintaining the alteration found in subsequent exams, and the other had the exam normalized in the third and final evaluation.

### 3.3. Radiological Evaluation

Of the 31 children included, 28 (90.3%) underwent some neuroimaging examination. Twenty-seven (87%) children underwent TFUS during their hospitalization. Twenty-six (96.3%) had normal results, and one (3.7%) showed abnormalities. Twenty-five children (80.6%) underwent brain MRI, and some also underwent computed tomography when the radiologist deemed it necessary to complement the examination. Seven children (28%) showed some alteration in the image. Table 1 shows the results of the radiological examinations of these patients.

### 3.4. Neurological Evaluation

In the evaluation performed by the neuropediatrician, 22 (81.5%) had the Gesell Neurodevelopmental Test applied between the ages of 12 and 27 months. Table 2 separately shows the neurological examination findings in the 22 patients who were evaluated between the ages of 18 and 27 months. Ten children (45.4%) presented some alteration in the examination. The most frequent alteration was language delay, which occurred in 31.8% of the cases, a suspected intellectual deficit in 9%, and hyperreflexia in 9%. All other findings, gait with hemiparesis, increased tone on one side, asymmetric posture, behavioral change with suspicion of autism spectrum disorder, microcephaly, global increase in tone, internal eversion of the feet during gait, clonus, opisthotonus, epilepsy, included thumbs, sleep disorder, and irritability in 4.5% of cases each. The most serious findings were found in the microcephalic patient.

Neurological symptoms, such as language delay, intellectual deficits, hyperreflexia, and microcephaly, appeared more prominently in children whose mothers were infected during the first trimester of pregnancy. There was a statistically significant difference in neurological findings in neurodevelopment between the trimesters of infection (*t*-test: 1st vs. 2nd trimester *p*-value = 0.01357; *t*-test: 1st vs. 3rd trimester *p*-value 0.03076).

## 4. Discussion

This study focuses on children with intrauterine exposure to the Zika virus who did not have microcephaly at birth, with no significant ophthalmological or auditory impairment. However, during outpatient follow-up of these children, it was observed that 50% of them presented some alteration in brain imaging, neurological examination, and/or neurodevelopment. The global delay in neurodevelopment of the only microcephalic patient was considered severe, and the findings in the other patients were considered mild in their absolute majority.

None of the children in the present study developed microcephaly after birth. Different findings were observed by Linden et al. [14] in the state of Ceará, northeast Brazil, a region heavily affected by the ZIKV epidemic. In a retrospective study of 13 children with normal head circumference at birth, they observed deceleration of head growth at 5 months of age and 11 of them were classified as having microcephaly. In this same study, however, all children presented abnormalities in radiological findings consistent with congenital Zika syndrome, including decreased brain volume, ventriculomegaly, subcortical calcifications, and cortical malformations, showing the severity of neurological impairment in the sample studied.

The findings of this study demonstrated that children born to mothers with confirmed ZIKV infection during pregnancy predominantly had normal hearing, with intact auditory neural pathways and hearing thresholds. A long-term hearing assessment was performed, including evaluations in the neonatal period and between 18 and 30 months. Only one child exhibited delayed absolute wave latencies in the neurodiagnostic BAEP, which was attributed to chronic middle ear effusion and not ZIKV exposure. These findings align with previous studies, such as those of Fandiño-Cárdenas et al. and Leite et al. [15,16], which reported preserved cochlear function and minimal hearing impairment among exposed children.

In ophthalmological assessments, 6.4% of children had optic nerve abnormalities at the end of the first year of life. Similar findings were noted in Zin et al. [17], where ocular abnormalities, particularly involving the optic nerve and retina, were frequent in children with congenital ZIKV infection, even in the absence of microcephaly or CNS anomalies. This highlights the importance of specialist evaluations for all exposed children, regardless of the stage of pregnancy or CNS findings. Although the microcephalic child in our study had normal retinal mapping, cortical visual impairment remains a possibility, as previously noted by Ventura and Lawrence [18], who emphasized universal visual impairment in children with SZC, irrespective of structural eye abnormalities.

Auditory and visual deficits may be related to structural damage to the inner ear or optic nerve. In murine models, as described by Cugola et al. [19], ZIKV has been shown to impact retinal development and cochlear structures. Additionally, functional tests in animals, such as auditory brainstem responses (ABRs) and optical coherence tomography (OCT), provide insights into the structural or functional origins of these deficits [20].

Consistent with prior studies, our findings highlighted characteristic radiological abnormalities in microcephalic patients, including reduced brain volume, cortical development anomalies, corpus callosum changes, ventriculomegaly, and calcifications. These results align with data from studies in Colombia [8], underscoring the consistent imaging profile associated with ZIKV-related microcephaly. Since the beginning of the microcephaly epidemic, studies have been published showing a characteristic radiological profile in patients with ZIKV. A study conducted in the state of Paraíba, northeast Brazil, described the results of MRI and/or CT scans of the skulls of fetuses and newborns with confirmed or strongly suspected ZIKV infection. The most notable change in the brain parenchyma described in all neonatal images was reduced brain volume. Abnormalities in cortical development associated with volume changes were observed in almost all cases analyzed, as well as abnormalities in the corpus callosum, ventriculomegaly, changes in cerebral gyrification, calcifications, and changes in the brain stem and corpus callosum [21]. Sanz Cortes et al. also described the same findings in a study conducted in Colombia [8].

It is important to highlight the presence of abnormalities in the brain imaging of children in this study that do not align with the typical classification of Congenital Zika Syndrome. These findings draw attention to the broader spectrum of radiological signs potentially associated with congenital ZIKV infection, particularly in children without microcephaly and with milder clinical signs and symptoms. This observation aligns with other studies on the follow-up of children with intrauterine exposure to ZIKV [22,23], although such studies remain limited in number and scope.

The calcifications and other central nervous abnormalities observed in Congenital Zika Syndrome (CZS) may be explained by biological processes such as neuronal loss, microglial activation, or inflammation resolution. Studies in animal models, such as those by Souza et al., demonstrated that ZIKV preferentially infects neural progenitor cells, causing apoptosis and calcifications because of tissue repair processes [24].

In the present study, 50% of the children studied presented some alteration in the brain imaging, neurological examination, or the Gesell Neurodevelopment Test. Premature children were evaluated for neurodevelopment according to the corrected gestational age. However, it is impossible to rule out that some delay in the child development milestone in this specific group may be associated with prematurity. A similar result could be observed in the study by Faiçal et al. [9], who studied 29 normocephalic children with intrauterine exposure to the Zika virus and described neurodevelopmental delay in 35%, with language delay in 31%, cognitive delay in 4%, and motor delay in 3% of the children evaluated using the Bayley scale of infant neurodevelopment. The same can be observed by Brasil, Lopes Moreira, and Nielsen-Saines [2,22,25], who, in a study to evaluate neurodevelopment in children exposed to ZIKV, described changes in at least one of the assessment areas of the Bayley scale in 37.2% of the children submitted to evaluation (25.5% of them between 1SD and 2SD of normality and 11.7% below 2SD of normality). Brasil, Lopes Moreira, and Nielsen-Saines [2,22,25] also describe in the same study cognitive changes in 11.7% of the children evaluated, language in 26.6%, and motor in 19.1% of the cases. Language delay was the most commonly affected area of child development in the present study and in the aforementioned scientific publications. These results are also compatible with the work published by Nielsen-Saines and collaborators [25], who evaluated neurodevelopment in 223 children with intrauterine exposure to the Zika virus and found neurodevelopmental delay in 28.7% of the assessed children and 3.7% of microcephalic children, once again showing that not only patients who presented microcephaly at birth are at risk of complications and sequelae.

We draw attention in the present study to a normocephalic child (number 9), whose radiological investigation performed through CT and MRI of the brain showed subcortical calcifications in the parietal lobes and predominantly in the frontal lobes, and a slight reduction in the volume of the cerebral gyri. This patient developed asymmetric gait due to left hemiparesis, again drawing attention to possible sequelae in patients with a head circumference within the normal range at birth. Other findings, such as suspected intellectual deficit and behavioral changes with suspected autism spectrum disorder, were also observed in the present study. The same was reported in the study by Nielsen-Saines et al. [25], who observed a frequency of 2.1% of autism spectrum disorder in 2-year-old children exposed to ZIKV during pregnancy.

One of the limitations of our study is the small number of children included. This is because we adopted laboratory confirmation of Zika virus infection as the inclusion criterion in this study. Since the viremia period is short, most pregnant women were outside the ideal period for collecting biological material for the conclusive diagnostic test, RT-PCR. However, although many cohorts describe a larger sample, few are as rigorous in selecting cases, with many lacking proper laboratory confirmation. Another limitation is that we did not have a control group to guarantee more accuracy in the results. We also cannot confirm that the exposed fetuses were actually infected by the Zika virus, which is a limitation in the vast majority of studies, given the difficulty in diagnosing the disease in the neonatal period. Finally, it is impossible to rule out that the alterations found in the four premature infants in our study may be related solely to prematurity.

## 5. Conclusions

This study demonstrated that newborns of mothers proven to be infected with the Zika virus during pregnancy may present varying degrees of visual, auditory, and neurological impairment associated or not with microcephaly. However, the most severe neurological findings appear to be associated with microcephaly at birth and early maternal infection. Congenital ZIKV infection is undoubtedly a challenging disease for researchers and health professionals, mainly due to the number of uncertainties involved. Many factors related to its pathogenesis and the potential for impairment, especially in non-microcephalic newborns, have not yet been clarified, requiring more studies to clarify these points further.

## Figures and Tables

**Table 1:** Results of radiological examinations of newborns included in this study during the first year of life.

Patients	TFUS	Brain MRI and/or CT Scan
1	Normal	Normal
2	Normal	Normal
3	Normal	**Slight increase in the anterior subarachnoid space, compatible with the age**
4	N/P	Normal
5	Normal	Normal
6	Normal	Normal
7	Normal	Normal
8	Normal	N/P
9	Normal	**Subcortical calcifications in the parietal lobes and predominantly in the frontal lobes, arranged in an elongated, band-like fashion, following the cortical gyri, noting apparent cortical retraction and a slight reduction in the volume of some of these gyri.**
10	Normal	**Questionable reduction in the thickness of the right genicular–calcarine tract, cysts in the choroid plexus on the right, the two largest measuring 1.1 cm × 0.8 cm and 0.5 cm × 0.3 cm.**
11	Normal	N/P
12	Normal	Normal
13	Normal	**White matter in the process of myelination, with triangular areas of hyperintensity noted near the ventricular atria in the terminal myelination zones, more evident than usual.**
14	Normal	Normal
15	Normal	Normal
16	Normal	Normal
17	Normal	**Slight enlargement of the frontal peri encephalic CSF space. Slight increase in the frontal CSF space. Slightly ectatic lateral ventricles, somewhat more evident in the frontal extensions.**
18	Normal	Normal
19	Normal	**Linear calcification in the right putamen, observed on CT, not identified in the brain MRI study on the same day. Discrete perivascular spaces prominent in the peritrigonal regions.**
20	Normal	**Possible calcifications in frontal lobes, delayed operculation, increased periencephalic fluid.**
21	Normal	Normal
22	Note 1	**Microcephaly. Significant underdevelopment of the cerebral lobes, especially the frontal lobes, with significant oligogyria. Numerous calcifications affecting both cerebral hemispheres, most prominent in the frontal lobes, where they assume a linear arrangement, predominantly affecting the subcortical region. Significantly tapered corpus callosum. Enlarged lateral ventricles, especially in the posterior extensions (colpocephaly).**
23	N/P	N/P
24	Normal	Normal
25	Normal	Normal
26	Normal	Normal
27	Normal	Normal
28 *	N/P	N/P
29 *	Normal	N/P
30 *	Normal	Normal
31 *	N/P	N/P

Note 1: Technical difficulty in performing the exam, by reduced anterior fontanelle. Calcifications in both superficial hemispheres. Enlargement of the lateral ventricles. TFUS: transfontanellar ultrasound; MRI: magnetic resonance image; CT: computed tomography; N/P: not performed. * Outpatient follow-up losses.

**Table 2:** Neurological findings in neurodevelopment evaluation between 18 and 27 months of age.

Patients	Neurological Findings	Deep Reflexes	Tonus Abnormalities	Signs of Ataxia	Dyskinesia	Posture
1	**Suspected intellectual disability, language delay**	Normal	Absent	Absent	Absent	Normal
2	Absent	Normal	Absent	Absent	Absent	Normal
3	Absent	Normal	Absent	Absent	Absent	Normal
4	**Language delay**	Normal	Absent	Absent	Absent	Normal
5	**Language delay**	Normal	Absent	Absent	Absent	Normal
6	**Language delay**	Normal	Absent	Absent	Absent	Normal
7	**Suspected intellectual disability, language delay**	Normal	Absent	Absent	Absent	Normal
8	Absent	Normal	Absent	Absent	Absent	Normal
9	**Gait with left hemiparesis**	**Hyperreflexia of the left lower limb**	**Increased tone in the left lower limb**	Absent	Absent	**Asymmetrical**
10	Not evaluated					
11	Absent	Normal	Absent	Absent	Absent	Normal
12	Absent	Normal	Absent	Absent	Absent	Normal
13	**Internal eversion of the feet during walking**	Normal	Absent	Absent	Absent	Normal
14	Not evaluated					
15	Absent	Normal	Absent	Absent	Absent	Normal
16	Absent	Normal	Absent	Absent	Absent	Normal
17	**Language delay**	Normal	Absent	Absent	Absent	Normal
**18**	**Behavioral change, Suspected autism spectrum disorder**	Normal	Absent	Absent	Absent	Normal
19	Absent	Normal	Absent	Absent	Absent	Normal
20	Absent	Normal	Absent	Absent	Absent	Normal
21	Absent	Normal	Absent	Absent	Absent	Normal
22	**Microcephaly, epilepsy, double hemiparesis, language delay, sleep disorder, irritability**	**Hyperreflexia**	**Overall increase in tonus**	**Absent**	**Clonus**	**Opisthotonus and included thumbs**
23	Not evaluated					
24	Not evaluated					
25	Absent	Normal	Absent	Absent	Absent	Normal
26	Not evaluated					
27	Absent	Normal	Absent	Absent	Absent	Normal
28 *	Not evaluated					
29 *	Not evaluated					
30 *	Not evaluated					
31 *	Not evaluated					

* Outpatient follow-up losses.

## Data Availability

All data used in this study are described or summarized in the article’s tables and supplementary material. Other data may be made available upon reasonable request to the corresponding author.

## Supplementary Materials

The following supporting information can be downloaded at: https://www.mdpi.com/article/10.3390/v17020238/s1, Table S1: Laboratory investigation for ZIKV, Dengue, and Chikungunya in pregnant women and cord blood; Table S2: Clinical–epidemiological characteristics of pregnant women included in this study; Table S3: Clinical–epidemiological characteristics of pregnant women with ZIKV infection included in this study in a summarized form; Table S4: Serologies for TORCH group infections in pregnant women included in this study. Table S5: Serologies for TORCH group infections in newborns included in this study; Table S6: Clinical–epidemiological characteristics of newborns with intrauterine exposure to ZIKV; Table S7: Clinical–epidemiological characteristics of newborns with intrauterine exposure to ZIKV (*n*=31); Table S8: Results of hearing tests performed on children with intrauterine exposure to ZIKV (*n* = 31); and Table S9: Results of retinal mappings performed on children exposed to ZIKV during pregnancy (*n* = 31).

## Abbreviations

The following abbreviations are used in this manuscript:

BAEP	Brainstem Auditory Evoked Potentials
BAEP-a	Automated click BAEP
BAEP-neuro	Click BAEP for neurodiagnosis
BAEP-SF	Frequency-specific BAEP
CZS	Congenital Zika Syndrome
CT	Computed tomography
DICOM	Digital Imaging and Communications in Medicine
FLAIR	Fluid attenuated inversion recovery
FO	Fundoscopy
ICU	Intensive Care Unit
IgG	Immunoglobulin G
IgM	Immunoglobulin M
MRI	Magnetic Resonance Imaging
RNA	Ribonucleic acid
RT-PCR	Reverse transcriptase reaction followed by polymerase chain reaction
TEOAE	Transient Evoked Otoacoustic Emissions
TORCH	Toxoplasmosis, Other, Rubella, Cytomegalovirus, Herpes virus
ZIKV	Zika virus
