# Supplementary material for: Neurological, Radiological, Visual, and Auditory Findings in Children with Intrauterine Exposure to the Zika Virus
Source: Viruses. 2025 Feb 9;17(2):238. doi: 10.3390/v17020238 (PMC11861632; doi:10.3390/v17020238)
Supplement: Supplementary file 1 [file viruses-17-00238-s001.zip › viruses-3424292-supplementary.pdf]

Neurological, Radiological, Visual, and Auditory Alterations in Children with Intrauterine Exposure to the Zika Virus

Marlos Melo Martins, Andréa Bittencourt Guastavino, Maria Clara de Magalhães-Barbosa, Maria Helena de Magalhães-Barbosa, Cristiane Fregonesi Dutra Garcia, Bárbara Karine Gonet Amaral, Annamaria Ciminelli Barbosa, Halina Cidrini Ferreira, Jaqueline Rodrigues Robaina, Mariana Barros Genuino de Oliveira, Fernanda Freire Tovar-Moll, Roberto de Andrade Medronho, Antonio José Ledo Alves da Cunha, Joffre Amim Jr, and Arnaldo Prata-Barbosa.

Table S1. Laboratory investigation for ZIKV, Dengue, and Chikungunya in pregnant women and cord blood.

Pregnant women	ZIKV RT-PCR Serum	ZIKV RT-PCR Urine	ZIKV RT-PCR Placenta	ZIKV RT-PCR Yolk sac	ZIKV IgG/IgM Serum	ZIKV RT-PCR Cord blood	Dengue NS1	Dengue IgG/IgM	CHIKV RT-PCR
1	Pos	Neg	Neg	Neg	Pos/Neg	Neg	Neg	Pos/Neg	Neg
2	Pos	-	Neg	-	-	Neg	-	Pos/Pos	Neg
3	Pos	-	Neg	-	-	Neg	-	-	Neg
4	Neg	Neg	Pos	Pos	Neg/Neg	-	Neg	Pos/Neg	Neg
5	Pos	-	Pos	Pos	Pos/Neg	-	Neg	Pos/Neg	Neg
6	Pos	Pos	Neg	Neg	Pos/Neg	Pos	Neg	Pos/Neg	Neg
7	Pos	Neg	Neg	Neg	Pos/Neg	Neg	-	Pos/Neg	Neg
8	Pos	-	-	-	Pos/Neg	Neg	Neg	Pos/Neg	Neg
9	Pos	Neg	-	-	-	-	Neg	-	Neg
10	Pos	-	Neg	-	Pos/Neg	-	-	Neg/Neg	Neg
11	Neg	Pos	-	-	-	-	Neg	-	Neg
12	-	Pos	-	-	-	-	-	-	Neg
13	Pos	Neg	-	-	Pos/Neg	-	Neg	Pos/Neg	Neg
14	-	Pos	-	-	Pos/Neg	-	-	Pos/Neg	Neg
15	Pos	Neg	-	-	Pos/Neg	-	-	Pos/Neg	Neg
16	Neg	Pos	-	-	Pos/Neg	-	Neg	Pos/Neg	Neg
17	Pos	Pos	-	-	Pos/Neg	-	-	Pos/Neg	Neg
18	Pos	Pos	-	-	Pos/Neg	-	-	Pos/Neg	Neg
19	Pos	Neg	-	-	Pos/Neg	-	-	Pos/Neg	Neg
20	Pos	Neg	-	-	Pos/Neg	-	Neg	Pos/Neg	Neg
21	Pos	-	-	-	Neg/Pos	-	-	Pos/Neg	Neg
22	Pos	Pos	-	-	-	-	-	-	Neg
23	-	-	-	-	Neg/Pos	-	-	Pos/Neg	Neg
24	Pos	-	-	-	Pos/Neg	-	Neg	Pos/Neg	Neg
25	Pos	-	-	-	-	-	Neg	-	Neg
26	-	-	-	-	N/P/Pos	-	-	Neg/Neg	Neg
27	Pos	Pos	Neg	-	Pos/Neg	Neg	-	Pos/Neg	Neg
28	Pos	Neg	Neg	Pos	Pos/Neg	Pos	-	Pos/Neg	Neg
29	Neg	-	Neg	Pos	Pos/Neg	Neg	-	Pos/Neg	Neg
30	Pos	Neg	-	-	-	-	-	Pos/Neg	Neg
31*#	Neg	Neg	Neg	-	-	-	-	-	Pos
32*	-	-	-	-	-	Pos	-	-	Neg
33*	Pos	Neg	-	-	-	-	Neg	-	Neg

ZIKV: Zika VIRUS; RT-PCR: Reverse transcriptase reaction followed by polymerase chain reaction; IgG: Immunoglobulin G; IgM: Immunoglobulin M; NS1: non-structural antigen 1**; - : not performed**; CHIKV: Chikungunya virus; Pos: Positive; Neg: negative.

*Fetal death # RT-PCR for Zika confirmed in fetal tissue.

Table S2. Clinical-epidemiological characteristics of pregnant women included in the study.

Pregnant women	Age at delivery (years)	Gestational age at infection	Reason for inclusion in the study	Education	Race	Alcoholism	Smoking
1	34	2nd trimester	Exanthema	High School	Black (mixed)	No	No
2	33	3rd trimester	Exanthema	High School	Black (mixed)	No	No
3	35	2nd trimester	Exanthema	Elementary	White	No	No
4	24	3rd trimester	Exanthema	Middle School	White	No	No
5	22	2nd trimester	Exanthema	Elementary	White	No	No
6	30	2nd trimester	Exanthema	High School	White	No	No
7	34	2nd trimester	Exanthema	Middle School	White	No	Yes
8	26	1st trimester	Exanthema	Elementary	White	No	No
9	41	2nd trimester	Exanthema	Elementary	Black (mixed)	Yes	No
10	27	1st trimester	Exanthema	College	White	No	No
11	34	3rd trimester	Exanthema	High School	White	No	No
12	21	3rd trimester	Exanthema	High School	Black (mixed)	No	No
13	29	1st trimester	Exanthema	High School	White	Yes	No
14	19	1st trimester	Exanthema	High School	White	No	No
15	24	1st trimester	Exanthema	College	White	No	No
16	31	1st trimester	Exanthema	High School	Black (mixed)	No	No
17	29	2nd trimester	Exanthema	High School	White	No	No
18	35	3rd trimester	Exanthema	College	Black	No	No
19	39	2nd trimester	Exanthema	High School	Black (mixed)	No	No
20	26	2nd trimester	Exanthema	Elementary	Black (mixed)	No	No
21	28	1st trimester	**Microcephaly**	Elementary	Black (mixed)	No	No
22	21	3rd trimester	Exanthema	Middle School	Black (mixed)	No	No
23	19	2nd trimester	Exanthema	Middle School	White	No	No
24	31	1st trimester	Exanthema	Middle School	Black	No	No
25	19	3rd trimester	Exanthema	Middle School	Black (mixed)	No	No
26	31	1st trimester	Exanthema	Middle School	White	No	No
27	34	2nd trimester	Exanthema	Middle School	White	No	No
28	22	3rd trimester	Exanthema	Middle School	Black (mixed)	No	No
29	24	2nd trimester	Exanthema	Middle School	Black (mixed)	No	No
30	21	1st trimester	Exanthema	Middle School	White	No	No
31*	19	2nd trimester	Exanthema	Middle School	Black (mixed)	Yes	No
32*	29	2nd trimester	**Microcephaly**	College	White	No	No
33*	23	1st trimester	Exanthema	High School	White	No	No

*Fetal death

Table S3. Clinical-epidemiological characteristics of pregnant women with ZIKV infection included in the study in a summarized form.

**Age at delivery (years)** [median (IQR)]	28 (22-33)
**Trimester of infection** [n (%)]	
1st trimester	11 (33.3%)
2nd trimester	14 (42.4%)
3rd trimester	8 (24.3%)
**Reason for inclusion in the study** [n (%)]	
Exanthema	31 (93.9%)
Microcephaly in obstetric ultrasound	2 (6.1%)
**Abortion** [n (%)]	3 (9.1%)
**Education** [n (%)]	
Elementary school	6 (18.2%)
Middle school	12 (36.4%)
High School	11 (33.3%)
College/University	4 (12.1%)
**Race** [n (%)]	
White	18 (54.6%)
Black (mixed)	13 (39.4%)
Black	2 (6%)
Asian	0 (0%)
Indian	0 (0%)
**Alcoholism in pregnancy** [n (%)]	3 (9.1%)
**Smoking in pregnancy** [n (%)]	1 (3%)
**Drug addiction in pregnancy** [n (%)]	0 (0%)

ZIKV: Zika VIRUS; IRQ: Interquartile range.

Table S4. Serologies for TORCH group infections in pregnant women included in the study.

Pregnant women	Toxoplasmosis (IgG/IgM)	Rubella (IgG/IgM)	CMV (IgG/IgM)	Herpes (IgG/IgM)	VDRL	HIV
1	-	-	-	-	Negative	Negative
2	Pos/Neg	Pos/Neg	Pos/Neg	Pos/Ind	Negative	Negative
3	Pos/Neg	Pos/Neg	Pos/Neg	Pos/Neg	Negative	Negative
4	Neg/Neg	Neg/Neg	Pos/Neg	Pos/Neg	Negative	Negative
5	Pos/Neg	Pos/Neg	Pos/Neg	Pos/Neg	Negative	Negative
6	Pos/Neg	Pos/Neg	Pos/Neg	Pos/Neg	Negative	Negative
7	-	-	-	-	Negative	Negative
8	Pos/Neg	Pos/Neg	Pos/Neg	Pos/Neg	Negative	Negative
9	Pos/Neg	Pos/Neg	Pos/Neg	Pos/Neg	Negative	**Positive**
10	Pos/Neg	Pos/Neg	Pos/Neg	Pos/Neg	Negative	Negative
11	Pos/Neg	Pos/Neg	Pos/Neg	Pos/Neg	Negative	Negative
12	Pos/Neg	-	-	-	Negative	Negative
13	Neg/Neg	Pos/Neg	Pos/Neg	Pos/Neg	Negative	Negative
14	Pos/Neg	Pos/Neg	Pos/Neg	Pos/Neg	Negative	Negative
15	Neg/Neg	Pos/Neg	Pos/Neg	N/P/N/P	Negative	Negative
16	Pos/Neg	Pos/Neg	Pos/Neg	Pos/Neg	Negative	Negative
17	Pos/Neg	Pos/Neg	Pos/Neg	Pos/Neg	Negative	Negative
18	Neg/Neg	-	-	-	Negative	Negative
19	Pos/Neg	Pos/Neg	Pos/Neg	Pos/Neg	Negative	Negative
20	Pos/Neg	-	-	-	Negative	Negative
21	Pos/Neg	Pos/Neg	Neg/Neg	Pos/Neg	Positive	Negative
22	Pos/Neg	Pos/Neg	Pos/Neg	Pos/Neg	Negative	Negative
23	Neg/Neg	Pos/Neg	-	Pos/Neg	**Positive**	Negative
24	Neg/Neg	-	-	-	Negative	Negative
25	Pos/Neg	Pos/Neg	Pos/Neg	Pos/Neg	Negative	Negative
26	Neg/Neg	N/P/Neg	-	Neg/Neg	Negative	Negative
27	Pos/Neg	Pos/Neg	Pos/Neg	-	Negative	Negative
28	-	-	-	-	Negative	Negative
29	Pos/Neg	Pos/Neg	-	-	Negative	Negative
30	Pos/Neg	Pos/Neg	Pos/Neg	Neg/Ind	Negative	Negative
31*	Pos/Neg	Pos/Neg	Pos/Neg	Pos/Neg	Negative	Negative
32*	Pos/Neg	Pos/Neg	Pos/Neg	Pos/Neg	Negative	Negative
33*	-	Pos/Neg	Pos/Neg	-	Negative	Negative

TORCH: Toxoplasmosis, other, rubella, cytomegalovirus, herpes'CMV: cytomegalovirus; HIV: human immunodeficiency virus; **VDRL: (Venereal Disease Research Laboratory) nontreponemal serologic test for the diagnosis of syphilis;** IgG: immunoglobulin G; IgM: immunoglobulin M; **- : Not performed;** Pos: Positive; Neg: negative; Ind: indeterminate.

*Fetal death

Table S5. Serologies for TORCH group infections in newborns**, in peripheral blood on the fifth day of life,** included in the study.

**Newborn**	**Toxoplasmosis (IgG/IgM)**	**Rubella (IgG/IgM)**	**CMV (IgG/IgM)**	**Herpes (IgG/IgM)**	**VDRL**	**HIV**
1	Pos/Neg	Pos/Neg	Pos/Neg	Pos/Neg	-	-
2	Pos/Neg	Neg/Neg	Pos/Neg	Pos/Neg	-	-
3	Neg/Neg	Pos/Neg	Pos/Neg	Pos/Neg	-	-
4	-	-	-	-	-	-
5	Pos/Neg	Pos/Neg	Pos/Neg	Neg/Neg	-	-
6	Pos/Neg	Pos/Neg	Pos/Neg	Neg/Neg	-	-
7	Pos/Neg	Pos/Neg	Pos/Neg	Pos/Neg	-	-
8	-	-	-	-	-	-
9	Pos/Neg	Pos/Neg	Pos/Neg	Pos/Neg	-	-
10	Pos/Neg	Pos/Neg	Pos/Neg	Pos/Neg	-	**Positive**
11	Neg/Neg	Pos/Neg	Pos/Neg	Pos/Neg	-	-
12	Neg/Neg	Pos/Neg	Pos/Neg	Neg/Neg	-	-
13	Neg/Neg	Pos/Neg	Pos/Neg	Neg/Neg	-	-
14	Neg/Neg	Pos/Neg	Pos/Neg	Neg/Neg	-	-
15	Neg/Neg	Pos/Neg	Pos/Neg	Pos/Neg	-	-
16	Neg/Neg	Pos/Neg	Pos/Neg	Pos/Neg	-	-
17	Pos/Neg	Pos/Neg	Pos/Neg	Pos/Neg	-	-
18	Pos/Neg	Pos/Neg	Pos/Neg	Pos/Neg	-	-
19	Neg/Neg	Pos/Neg	Pos/Neg	Pos/Neg	-	-
20	-	-	-	-	-	-
21	Pos/Neg	Pos/Neg	Neg/Neg	Pos/Neg	-	-
22	Pos/Neg	Pos/Neg	Neg/Neg	Pos/Neg	Negative	-
23	-	-	-	-	-	-
24	Neg/Neg	Pos/Neg	Pos/Neg	Pos/Neg	Negative	-
25	Neg/Neg	Pos/Neg	Pos/Neg	Pos/Neg	-	-
26	-	-	-	-	-	-
27	Neg/Neg	Pos/Neg	Pos/Neg	Pos/Neg	-	-
28	-	-	-	-	-	-
29	Neg/Neg	Pos/Neg	Pos/Neg	Pos/Neg	-	-
30	Pos/Neg	Pos/Neg	Pos/Neg	Pos/Neg	-	-
31	-	-	-	-	-	-

TORCH: Toxoplasmosis, other, rubella, cytomegalovirus, herpes; CMV: cytomegalovirus; HIV: human immunodeficiency virus; **VDRL: (Venereal Disease Research Laboratory) nontreponemal serologic test for the diagnosis of syphilis;** IgG: immunoglobulin G; IgM: immunoglobulin M**; - : Not performed**; Pos: Positive; Neg: negative.

Table S6. Clinical-epidemiological characteristics of newborns with intrauterine exposure to ZIKV.

Newborn	Gestational age (weeks + days)	Sex	Weight (grams)	HC (cm)	Weight x gestational age	Microcephaly	Other malformations	NICU
1	32	M	1620	28.5	AGA	No	No	**Yes**
2	38+2	F	2670	34.0	AGA	No	No	No
3	37	F	2859	33.5	AGA	No	No	No
4	39	F	3395	35.0	AGA	No	No	No
5	32+1	F	1905	30.0	AGA	No	No	**Yes**
6	32+1	F	1605	31,5	AGA	No	No	**Yes**
7	41+5	M	3540	34.0	AGA	No	No	No
8	40+4	F	3550	34.5	AGA	No	No	No
9	40+4	F	3570	36.0	AGA	No	No	No
10	41+1	M	3850	35.7	AGA	No	No	No
11	40+3	F	3750	35.0	AGA	No	No	No
12	39	M	2995	35.0	AGA	No	No	No
13	39	F	2870	35.0	AGA	No	No	No
14	40	F	3535	34.5	AGA	No	No	No
15	38	M	2160	31.7	**SGA**	No	No	No
16	39+5	F	3490	34.0	AGA	No	No	No
17	38+4	M	2785	34.0	AGA	No	No	No
18	38+5	F	3080	33.0	AGA	No	No	No
19	40+3	F	3340	33.5	AGA	No	No	**Yes**
20	38+4	F	3055	34.0	AGA	No	No	No
21	39+5	F	4130	36.0	**LGA**	No	No	No
22	39+3	F	2690	28.5	AGA	**Yes**	No	No
23	38+4	F	3355	33.5	AGA	No	No	No
24	36+3	F	2990	33.0	AGA	No	No	No
25	38+2	F	3220	35.0	AGA	No	No	No
26	39+2	F	3205	32.0	AGA	No	No	No
27	37+6	M	3200	36.0	AGA	No	No	No
28	38+2	F	3935	36.0	**LGA**	No	No	No
29	40+5	F	3315	33.0	AGA	No	No	**Yes**
30	41	M	2765	33.0	AGA	No	No	No
31	40+1	M	3975	36.0	AGA	No	No	No

ZIKV: Zika VIRUS; HC: head circumference; NICU: admission to the Neonatal Intensive Care Unit; F: female M: male; AGA: adequate for gestational age; SGA: small for gestational age; LGA: large for gestational age

Table S7. Clinical-epidemiological characteristics of newborns with intrauterine exposure to ZIKV (n=31).

**Gestational age at birth – in weeks [n (%)]**	
<28	0 (0%)
28-32	3 (9.7%)
33-36	1 (3.2%)
37-41	27 (87.1%)
> 42	0 (0.00)
**Sex [n (%)]**	
Female	22 (71%)
Male	9 (29%)
**Birth weight – in grams [n (%)]**	
< 1000	0 (0%)
1000-1500	0 (0%)
1500-2000	3 (9.7%)
2000-2500	1 (3.2%)
2500-3000	8 (25.8%)
> 3000	19 (61.3%)
**Weight for gestational age [n (%)]**	
SGA	1 (3.2%)
AGA	28 (90.3%)
LGA	2 (6.5%)
**Head circumference classification [n (%)]**	
Normocephalic	30 (96.8%)
Microcephalic	1 (3.2%)
**Other malformations [n (%)]**	0 (0%)
**Admission to the NICU [n (%)]**	5 (16.1%)
**Death in the neonatal period [n (%)]**	0 (0%)

ZIKV: Zika virus; AGA: adequate for gestational age; SGA: small for gestational age; LGA: large for gestational age; NICU: Neonatal Intensive Care Unit.

Table S8. Results of hearing tests performed on children with intrauterine exposure to ZIKV.

	**Hearing tests (exam date)**			
**Patients**	**TEOAE/PNA**	**BAEP-a/PNA**	**BAEP-neuro/PNA**	**BAEP-SF/PNA**
1	Passed, BE 03 days	-	**LE: Absolute latency delay** OE: Normal 10 days	-
2	Passed, BE 01 day	-	Normal, BE 03 days	Normal, BE 03 days
3	Passed, BE 01 day	-	Normal, BE 09 days	Normal, BE 09 days
4	Passed, BE 06 days	U/R	Normal, BE 1 year 10 months	Normal, BE 1 year 10 months
5	Passed, BE 19 days	Normal, BE 30 days	-	-
6	Passed, BE 19 days	Normal, BE 31 days	-	-
7	Passed, BE 02 days	Normal, BE 23 days	-	-
8	Passed, BE 02 days	Normal, BE 7 months	-	-
9	Passed, BE 1 month	Normal, LE; U/R, RE 5 months	Normal, BE 8 months	Normal, BE 8 months
10	Passed, BE 01 day	Normal, LE; U/R, RE 23 days	-	-
11	Passed, BE 02 days	-	-	-
12	Passed, BE 06 days	Normal, BE 4 months	-	-
13	Passed, BE 01 day	-	Normal, BE 9 months	Normal, BE 9 months
14	Passed, BE 01 day	U/R	-	-
15	Passed, BE 02 days	U/R	Normal, BE 4 months	Normal, BE 4 months
16	Passed, BE 03 days	Normal, BE 5 months	-	-
17	Passed, BE 01 day	Normal, BE 2 months	-	-
18	Passed, BE 01 day	Normal, BE 2 months	-	-
19	Passed, BE 02 days	-	Normal, BE 1 year 4 months	Normal, BE 1 year 4 months

20	Passed, BE<br>01 day	Normal, BE<br>2 months	-	-
21	Passed, BE<br>23 days	Normal RE;<br>U/R, LE<br>4 months	-	-
22	Passed, BE<br>01 day	Normal, BE<br>3 months	Normal, BE<br>1year 3 months	Normal, BE<br>1 year 3 months
23	Passed, BE<br>03 days	-	-	-
24	Passed, BE<br>03 days	-	Normal, BE<br>1 year 2 months	Normal, BE<br>1 year 2 months
25	Passed, BE<br>01 day	Normal, BE<br>2 months	Normal, BE<br>1 year	Normal, BE<br>1 year
26	Passed, BE<br>02 days	U/R	-	-
27	**Failed, LE**<br>04 days	Normal, LE; U/R, RE<br>11 days	Normal, BE<br>1 year	-
28*	Passed, BE<br>21 days	-	-	-
29*	Passed, BE<br>11 days	-	-	-
30*	Passed, BE<br>04 days	-	-	-
31*	Passed, BE<br>01 day	-	-	-

ZIKV: Zika virus; TEOAE: Transient Evoked Otoacoustic Emissions; BAEP: Brainstem Auditory Evoked potentials; BAEP-a: Automated click BAEP; BAEP-neuro: Click BAEP for neurodiagnosis; BEAP-SF: Frequency-specific BAEP; **PNA: Post natal age;** BE: both ears; LE: left ear; RE: right ear; **- : not performed**; U/R: unreliable result *Outpatient follow-up losses

Table S9. Results of retinal mappings performed on children exposed to ZIKV during pregnancy.

**Patients**	**1st exam**	**2nd exam**	**3rd exam**
1	Normal	Normal	Normal
2	Normal	Normal	Normal
3	Normal	Normal	Normal
4	Normal	-	-
5	Normal	Normal	Normal
6	Normal	Normal	Normal
7	Normal	Normal	-
8	Normal	Normal	Normal

9	Normal	Normal	Normal
10	Normal	-	-
11	Normal	Normal	Normal
12	**Increased cupping of the optic nerve, both eyes**	**Increased cupping of the optic nerve, both eyes**	**Increased cupping of the optic nerve, both eyes**
13	Normal	Normal	-
14	Normal	Normal	-
15	Normal	Normal	Normal
16	Normal	-	-
17	Normal	Normal	-
18	Normal	-	Normal
19	**Small and symmetrical optic nerve, both eyes**	**Small and symmetrical optic nerve, both eyes**	**Small and symmetrical optic nerve, both eyes**
20	Normal	Normal	Normal
21	Normal	Normal	-
22	Normal	Normal	Normal
23	Normal	-	-
24	Normal	Normal	Normal
25	**Right eye: Normal; Left eye: oval optic nerve; peridiscal hyperpigmentation**	**Retinal hemorrhage in left eye**	Normal
26	Normal	-	-
27	Normal	Normal	Normal
28*	-	-	-
29*	Normal	-	-
30*	Normal	-	-
31*	Normal	-	-

ZIKV: Zika vírus; **- : not performed.**

*Outpatient follow-up losses
